# The structure of neurofibromin isoform 2 reveals different functional states

**DOI:** 10.1038/s41586-021-04024-x

**Published:** 2021-10-27

**Authors:** Andreas Naschberger, Rozbeh Baradaran, Bernhard Rupp, Marta Carroni

**Affiliations:** 1 SciLifeLab, Department of Biochemistry and Biophysics, Stockholm University, Solna, Sweden; 2 Institute of Genetic Epidemiology, Medical University Innsbruck, Innsbruck, Austria; 3 k.-k. Hofkristallamt, San Diego, CA USA

**Keywords:** Growth factor signalling, Cryoelectron microscopy, Tumour-suppressor proteins

## Abstract

The autosomal dominant monogenetic disease neurofibromatosis type 1 (NF1) affects approximately one in 3,000 individuals and is caused by mutations in the *NF1* tumour suppressor gene, leading to dysfunction in the protein neurofibromin (Nf1)^1,2^. As a GTPase-activating protein, a key function of Nf1 is repression of the Ras oncogene signalling cascade. We determined the human Nf1 dimer structure at an overall resolution of 3.3 Å. The cryo-electron microscopy structure reveals domain organization and structural details of the Nf1 exon 23a splicing^3^ isoform 2 in a closed, self-inhibited, Zn-stabilized state and an open state. In the closed conformation, HEAT/ARM core domains shield the GTPase-activating protein-related domain (GRD) so that Ras binding is sterically inhibited. In a distinctly different, open conformation of one protomer, a large-scale movement of the GRD occurs, which is necessary to access Ras, whereas Sec14-PH reorients to allow interaction with the cellular membrane^4^. Zn incubation of Nf1 leads to reduced Ras-GAP activity with both protomers in the self-inhibited, closed conformation stabilized by a Zn binding site between the N-HEAT/ARM domain and the GRD–Sec14-PH linker. The transition between closed, self-inhibited states of Nf1 and open states provides guidance for targeted studies deciphering the complex molecular mechanism behind the widespread neurofibromatosis syndrome and Nf1 dysfunction in carcinogenesis.

## Abstract (web summary)

Cryo-EM structure of Nf1 protein is reported, revealing closed and open conformations that regulate interaction with Ras oncogene, setting the stage for understanding the mechanistic action of Nf1 and how disease mutations lead to dysfunction.

## Main

Nf1^5,6^ is a multifunctional tumour suppressor protein forming an obligate high-affinity dimer^7^ of about 640-kDa molecular weight. As a GTPase-activating protein (GAP), its primary function is suppression of the Ras signalling cascade by accelerating the GTP hydrolysis rate of Ras, which returns Ras to its inactive GDP bound form^4,8^. Consequently, mutated Nf1 shows altered Ras-GAP activity and leads to uncontrolled signalling in multiple cell signalling pathways. The resulting syndrome NF1 presents diverse phenotypes^9^, ranging from benign lesions to cognitive impairment and psychological retardation^1,2^. Patients with NF1 carry a higher overall lifetime risk for developing cancer. The *NF1* disease mutations are distributed over the entire protein^1,4^, with slightly higher occurrence in the catalytic GRD. About half of all cases of NF1 are inherited, whereas the remaining cases result from de novo mutations^10^. Somatic Nf1 mutations are also present in 5–10% of cancers, demonstrating the role of Nf1 as a tumour suppressor^1^.

The ubiquitous Nf1 isoform-2 splice variant Nf1-23a (2839aa) is, together with Nf1 isoform 1, which lacks the 23a insertion (2818aa), one of the two biologically relevant alternative Nf1 isoforms. Inclusion of exon 23a is found in most human tissues but predominately skipped in neurons of the central nervous system^3^, and variation of the isoform 1/2 splicing ratio leads to disturbed neuronal differentiation^11^. The 23a insertion, located within the GRD, leads to about tenfold less GAP activity^12^ than Nf1 isoform 1. The presence or absence of 23a regulates Ras/ERK signalling and affects memory and learning behaviour^12^.

Of the many proteins interacting with Nf1, only the interaction with Ras and Sprouty-related protein with an EVH1 domain (SPRED1) is well characterized^4,13^. SPRED1 recruits Nf1 from the cytosol to the plasma membrane where Ras resides, and Nf1 subsequently can downregulate GTP-bound Ras^14^.

## Domain organization of the Nf1-23a dimer

**Fig. 1:**
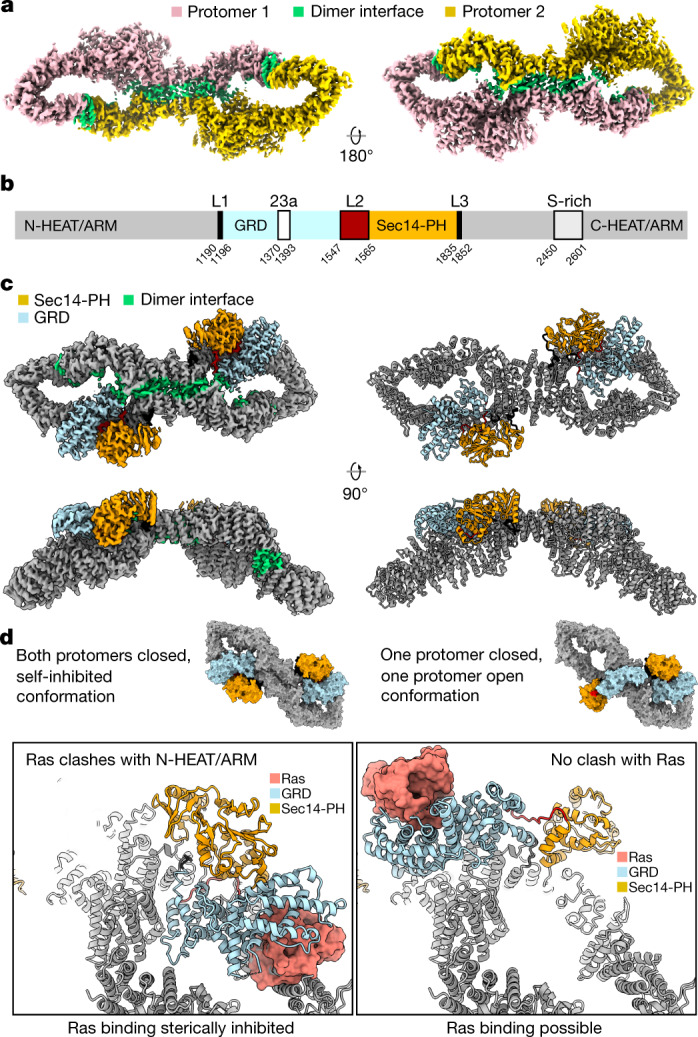
Structure of the neurofibromin homodimer. **a**, Overview of the Nf1 dimer with one protomer yellow and one pink. Dimer interaction regions are green. **b**, Domain organization of Nf1-23a. N-HEAT/ARM and C-HEAT/ARM domains are grey, GRD light blue and the Sec14-PH domain yellow. Locations of the interdomain linkers, exon 23a insert and S-rich region are annotated. **c**, Composite density map (left) and ribbon model (right) of the Nf1-23a homodimer in double-auto-inhibited conformation, coloured as in **b**. **d**, Nf1 double-auto-inhibited, closed state (left) and open state (right). The boxed insert (bottom left) shows that, in auto-inhibited, closed conformation, Ras, docked from the crystal structure complex with GRD (PDB entry 6ob3), cannot bind. In the open protomer conformation (right insert), GRD can bind Ras.

**Extended Data Fig. 1:**
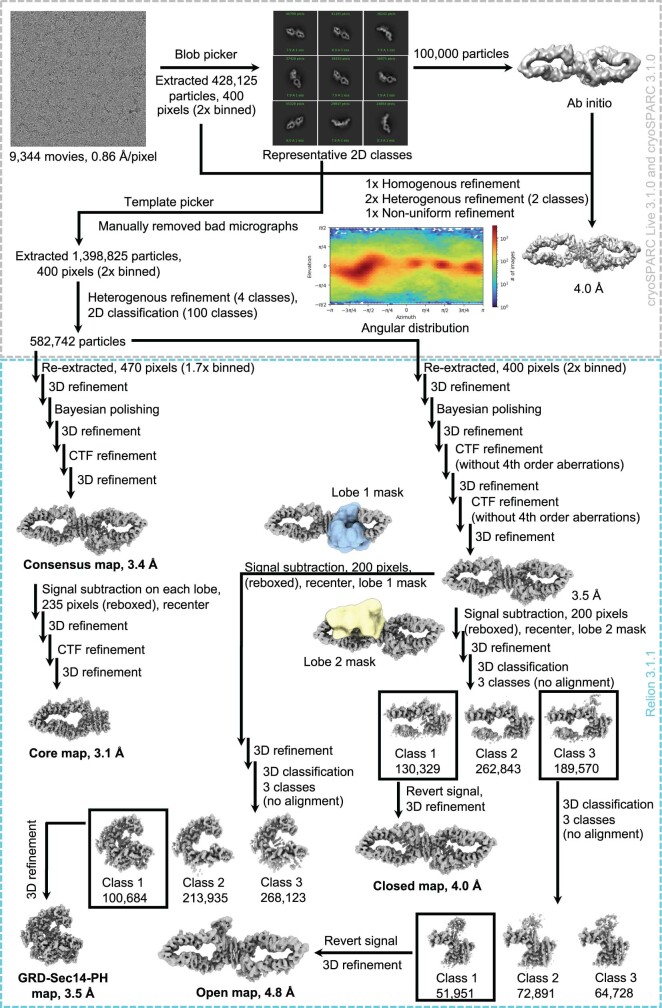
Flowchart illustrating cryo-EM data processing of native Nf1-23a in the absence of Zn. Details are described in the Methods section.

**Extended Data Fig. 2:**
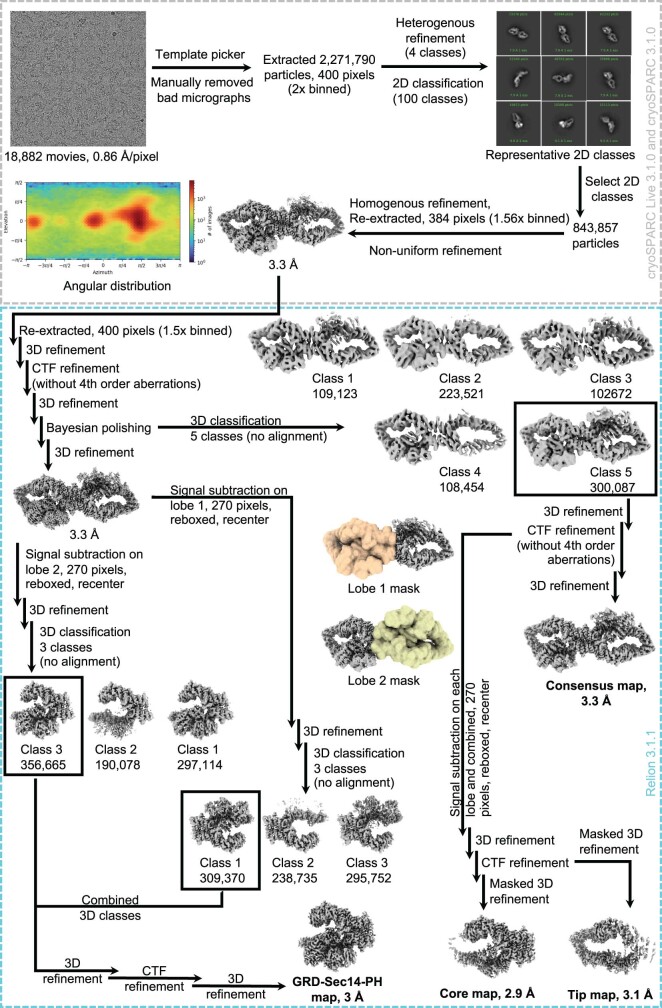
Flowchart illustrating cryo-EM data processing of Nf1-23a incubated with Zn. Details are described in the Methods section.

**Extended Data Fig. 3:**
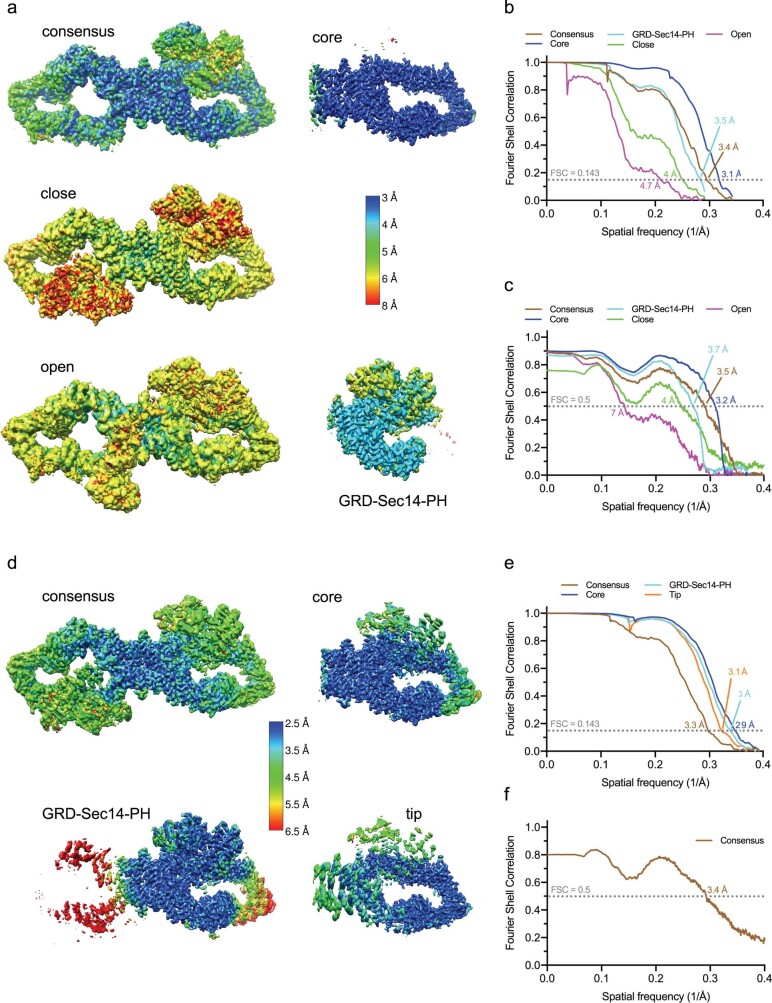
Local resolution of final reconstructions in the absence (a-c) and presence (d-f) of Zn. **a**, **d**, Final 3D reconstructions coloured according to local resolution estimation calculated by Resmap. **b**, **e**, Solvent-corrected “gold-standard” Fourier Shell Correlation (FSC) curves of the final 3D reconstructions. The estimated resolution (FSC = 0.143) for each reconstruction is indicated. **c**, **f**, Model-to-map FSCs. The estimated resolution (FSC = 0.5) for each model is indicated.

Our high-resolution single particle cryo-electron microscopy (EM) structures reveal the domain organization and structural details of the full-length Nf1-23a isoform 2 dimer (Fig. 1a–c, Extended Data Figs. 1–3). Contrasting an unpublished low-resolution, symmetric Nf1 model^15^, two distinct populations of the Nf1-23a dimer are present: (1) a major population with both protomers showing a closed, auto-inhibited conformation (closed Nf1 state) stabilized by Zn and (2) a second population (open Nf1 state) with one protomer in the auto-inhibited conformation and the other protomer in an open configuration necessary for Ras binding (Fig. 1d).

**Extended Data Fig. 4:**
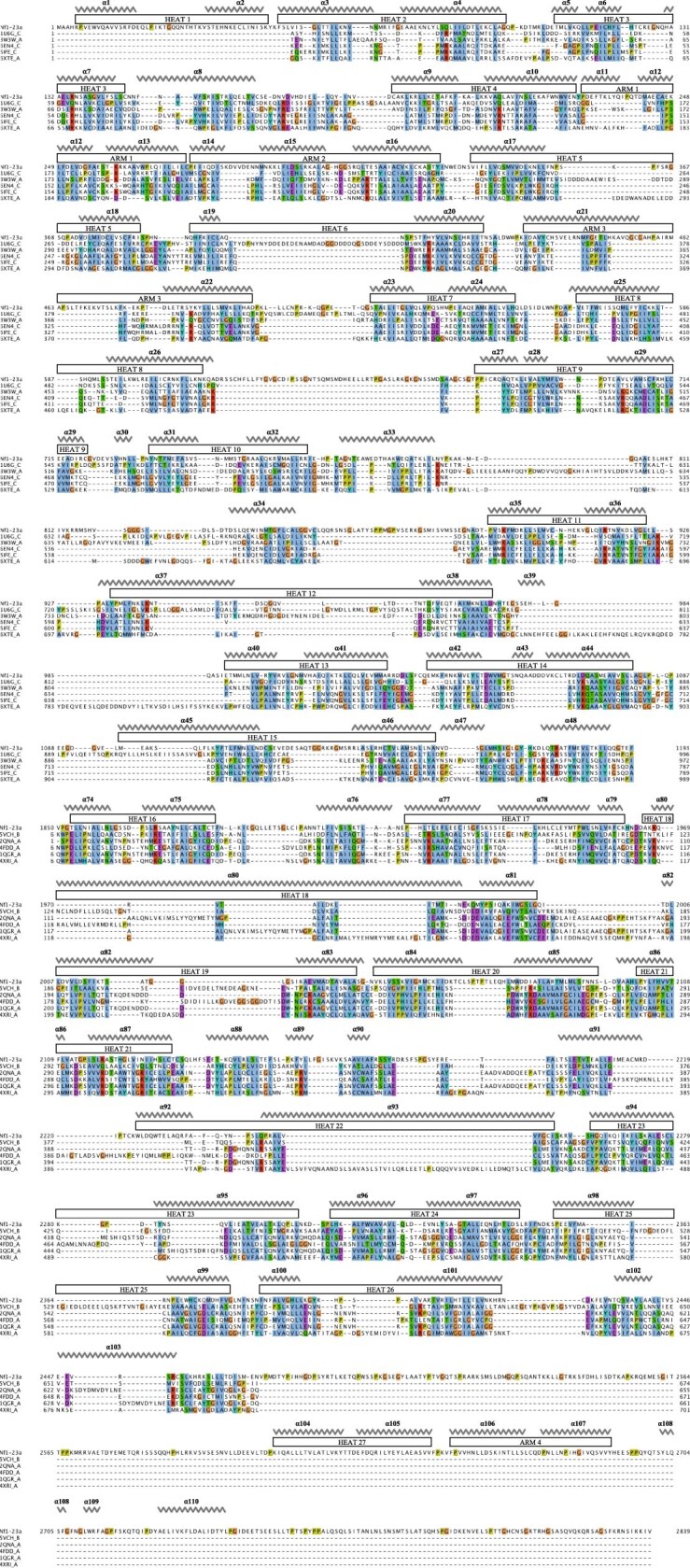
Structure-based sequence alignment of the core domains of Nf1-23a. Multiple sequence alignments of residues 1-1193 and 1850-2839 of Nf1-23a were performed in HHpred (https://toolkit.tuebingen.mpg.de/tools/hhpred) against PDB_mmCIF70_3_Mar and PDB_mmCIF30_3_Mar databases using default parameter settings with the exception of global alignment mode. The PDB codes and their corresponding chains that have aligned to Nf1-23a are shown. The amino acid sequences are coloured according to the ClustalW convention. Regions predicted to have HEAT- and ARM-repeats are indicated by rectangular boxes. The alpha-helices are highlighted by zig-zag grey lines and labelled according to Extended Data Fig. 6.

**Extended Data Fig. 5:**
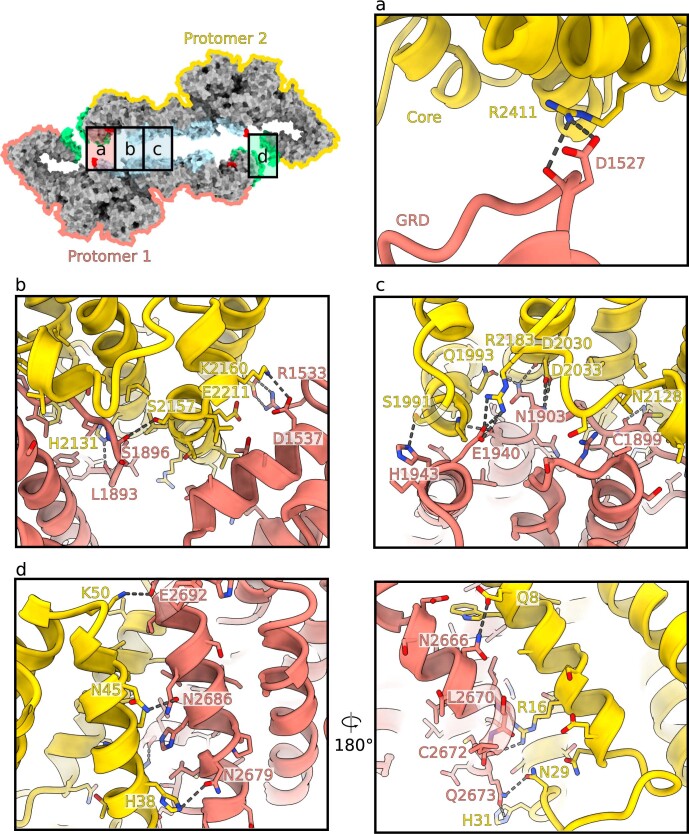
Details of Nf1 dimer interface interactions. Contact regions indicated in boxes (**a-d**) are detailed in the large panels. Participating protomer residues are coloured pink and yellow, respectively. Interactions are shown between **a**, GRD and C-HEAT/ARM core; **b**, **c**, the N-HEAT/ARM and C-HEAT/ARM core; **d**, the N-terminal tip of the N-HEAT/ARM domain and the C-terminal end of the C-HEAT/ARM domain. Hydrogen bonds/charged interactions are indicated by dashed lines.

An asymmetric, homodimeric core of 27 ordered HEAT-repeats and four ARM-repeats forms the molecular framework into which the GRD, including its non-catalytic GAPex subdomain^13^, and the membrane-associatedSec14-PH domain are centrally linked (Extended Data Fig. 4). Three major dimer interfaces between the protomers exist in both states. The N-terminal HEAT helices contact the terminal C-HEAT helices at the other protomer (Fig. 1a), each contact burying about 950 Å^2^ of surface area. The resulting dimer is connected in the centre, forming a core that buries almost 3,000 Å^2^ of surface area. The GRD and Sec14-PH domain extend out from the N-HEAT/ARM core, and the chain returns to the C-HEAT/ARM core near where it branched out. In the closed state, additional contacts between the last helix α63 of the GRD and the core bury another ~240 Å^2^ of surface. The linker between GRD and the Sec14-PH domain is in the closed conformation part of a Zn binding site, contributing a stabilising contact to the N-HEAT/ARM core. The dimer interfaces are detailed in Extended Data Fig. 5. The disordered, S-rich region branching out from C-HEAT helix α103 and returning into the core at C-HEAT helix α104 could not be traced. Flexibility and solvent access are consistent with the reported target for phosphorylation^4^.

## Open state

**Fig. 2:**
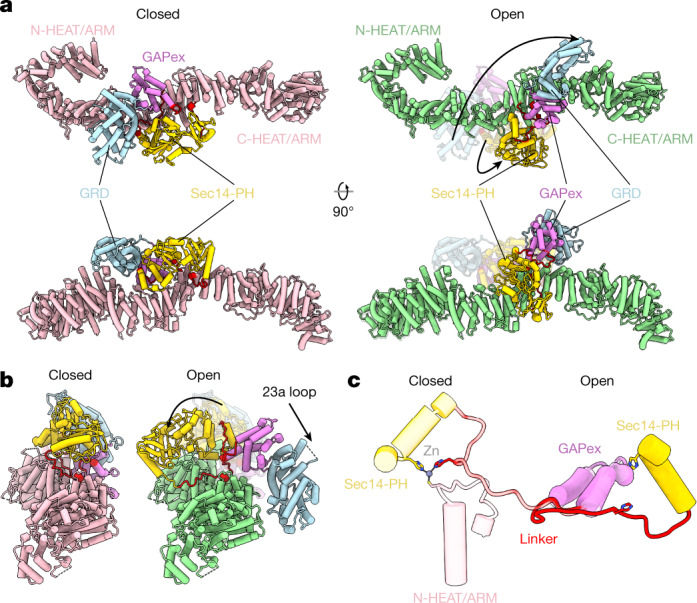
Conformational rearrangement between closed and open Nf1 states. **a**, Comparison of closed (pink, left panels) and open (green, right panels) conformation of the Nf1 protomers. Illustrated are the massive conformational rearrangement of the GRD (light blue), its GAPex domain (magenta) and its Sec14-PH domain (yellow). **b**, View of both states along the principal direction of the Nf1 dimer core. **c**, Detail of the Zn metal binding site stabilising the closed Nf1 conformation. Superposition of open and closed state on the last three helices of GAPex illustrates the large movement of the Sec14-PH domain against the GRD/GAPex domain.

**Extended Data Fig. 6:**
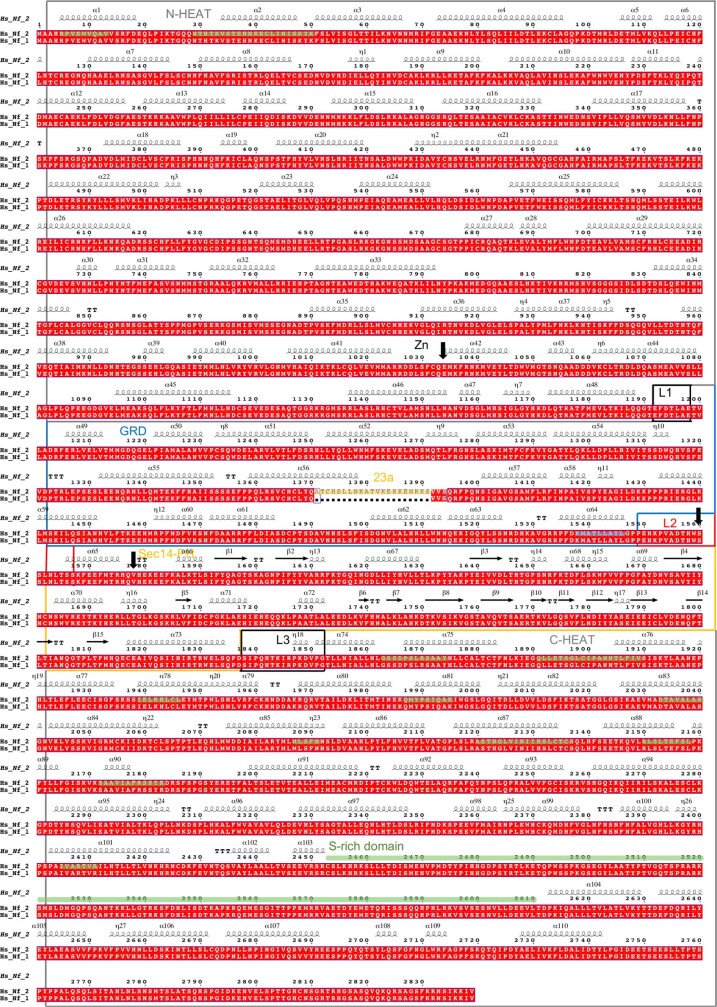
Sequence and model-based secondary structure assignment of neurofibromin isoform 2 (Nf1-23a) and neurofibromin isoform 1. Regions are boxed in grey for N-HEAT and C-HEAT domains, black for L1 and L3, red for L2, light blue for GRD and gold for Sec14-PH. The 21aa insertion in isoform 2 is highlighted in yellow. Metal-coordinating residues C1032, H1558, and H1576 are labelled with arrows. Contact regions between protomer termini and between the protomer cores are highlighted green, the contacts between GRD in closed conformation and the protomer core are highlighted cyan.

In the open Nf1 state, one protomer presents a closed conformation, where binding of Ras or a Ras dimer^16^ by the Nf1 GRD^17^ is sterically inhibited, with access of the Nf1 GRD to Ras completely blocked by the HEAT/ARM-repeat core. The conformation of the other protomer presents a distinctly different, open conformation, with the GRD and Sec14-PH domains reoriented and almost reversed in position compared to the closed conformation (Figs. 1d, 2a). The density maps reveal the linkages between the domains, including L1 between the N-HEAT/ARM and the GRD; the (in X-ray models absent) linker L2 between the GRD and the lipid-binding Sec14-PH domain; and L3, the linkage from Sec14-PH to the core C-HEAT/ARM domain (Extended Data Fig. 6). In the transition to the open conformation, these linkers undergo significant conformational rearrangement, which is crucial for the large-scale movement of the GRD necessary to bind Ras and for Sec14-PH to access the cellular membrane.

## Conformation changes between states

Supplementary Video 1 illustrates the rearrangement from the auto-inhibited, closed Nf1 state to the open state (Fig. 2a, b), in which Nf1 can access Ras and associate with the membrane. From the closed conformation blocking Ras access, the GRD and Sec14-PH domain rotate ~130º and ~90º, respectively, with the active Ras binding and the lipid binding site of the respective domains facing away from the core and facilitating membrane access.

Connecting loop L1 (G1190 to L1196) between the N-HEAT core helix α48 (L1173 to G1190) and GRD helix α49 (L1196 to M1215) rearranges completely and then forms an extension of the two almost aligned helices, with G1190 as a plausible hinge point. To accommodate relocation of the GRD, the Sec14-PH domain also undergoes a ~90º rotation, and its linker L3 to the C-HEAT/ARM domain completely changes conformation in proline-rich region Q1835 to G1852. The proximity of L1 and L3 to each other, at the centre of the overall rotation, is of fundamental importance, because modest local conformational changes suffice to accommodate the large rotation and long-range relocation of the two domains. Were L1 and L3 far distant from each other, both domains would remain clamped to the core, unable to undergo the large rotational rearrangement.

The relative movement of the GRD and Sec14-PH domain against each other also requires a conformational change of the long L2 interdomain linker. Extending from the last helix α63 of the GRD, the L2 linker hinges at a proline-rich loop beginning around G1547 (G1526 in Nf1 isoform 1 X-ray models) to T1565, continuing into a three-turn helix that becomes part of the Sec14 domain and connects with a short loop to the first helix α65 of the Sec14 domain (Extended Data Fig. 6). This connecting loop and the last helix α73 are not present in the Sec14-PH domain X-ray models.

## Metal binding site

**Fig. 3:**
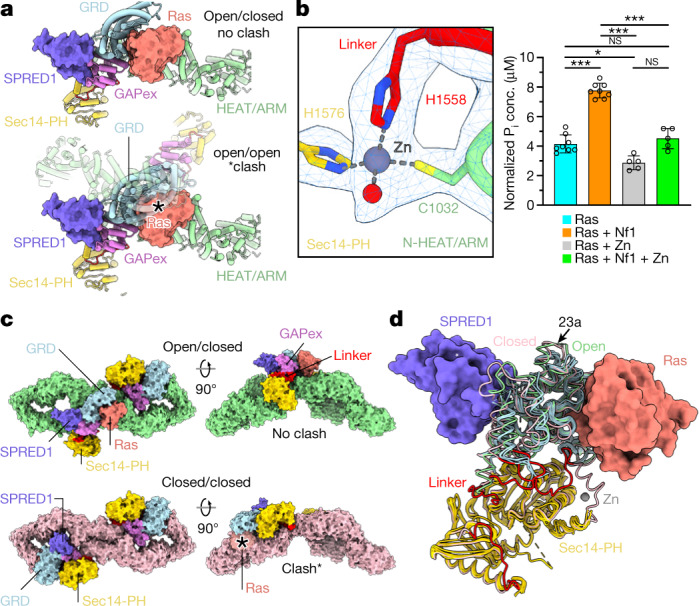
Zn site and conformation of Nf1 and SPRED1 binding. Colour scheme as in Fig. 2. **a**, A conformational state with both protomers in the open conformation was not observed and is impeded by severe steric interference (indicated by the asterick) between the two respective GRDs. **b**, Insert: density around the metal binding site, with tetrahedral His_2_Cys-water coordination and distances typical for Zn^2+^. Right panel: Nf1-23a accelerates the rate of GTP hydrolysis by KRas. Error bars represent the mean ± s.e.m. of *n* = 5 or *n* = 8 independent assays of the same sample as indicated in each bar by individual data points. Significance calculated by one-way ANOVA followed by pairwise two-sided *t*-tests, applying Bonferroni correction with α = 0.01. *P = 0.0016, ***P < 0.000052; NS, not significant. **c**, Top: concomitant SPRED1 and Ras binding is possible only to the protomer in open conformation. Bottom: In the closed conformation, SPRED1 can bind, whereas Ras cannot bind. **d**, Superposition of all available GRD and Sec14-PH domains onto the Protein Data Bank (PDB) model 1nf1. The backbone traces of GRD X-ray models are shown light blue, and the X-ray Sec14-PH domains are shown in yellow. The GRD loop C1486-D1490, not visible in the X-ray structures, must undergo rearrangement upon SPRED1 binding in both conformations because it interferes with bound SPRED1.

**Extended Data Fig. 7:**
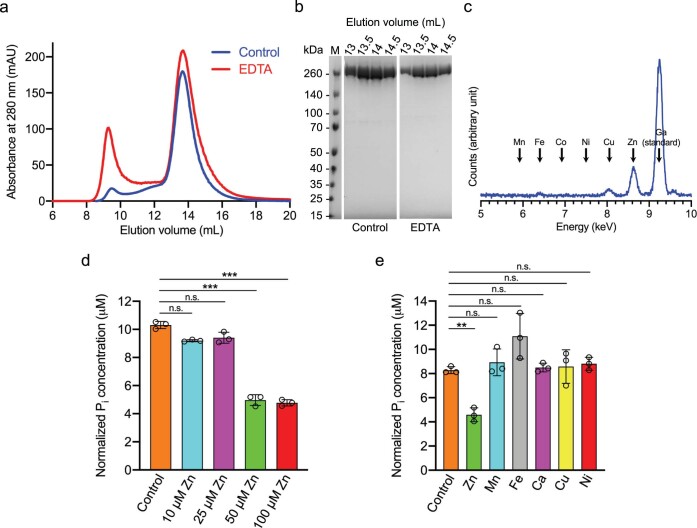
Purification, characterization, and activity assays of Nf1-23a. **a**, Size-exclusion chromatography purification of Nf1-23a in the absence (blue line) and presence (red line) of EDTA. In both preparations, Nf1-23a purified as a single peak, but with a higher void peak (~9 mL) in the presence of EDTA in comparison to without. **b**, SDS-PAGE analysis of corresponding fractions from the size-exclusion chromatography. Nf1-23a is highly pure by SDS-PAGE. For gel source data see Supplementary Figure 1. **c**, Total reflection X-ray fluorescence (TXRF) spectrum of purified Nf1-23a identified the presence of Zn in native Nf1. Measurements on both sample and buffer were done in presence of a gallium internal standard at 2 mg L^−1^, added to the samples (1:1, v/v) before measurements. Buffer subtracted difference spectrum normalized by internal standard. Kα X-ray emission lines for different metals are indicated with arrows. **d**, The effect of different concentration of Zn on the rate of Nf1-23a GTP hydrolysis by KRas. Each assay was measured with 3 independent replications. The concentration of P_i_ in each assay was normalized against both the buffer + Zn and Nf1 + Zn control. Error bars represent the mean ± s.e.m. of n = 3 independent assays of the same sample. Significance calculated by one-way ANOVA followed by pairwise 2-sided t-tests, applying Bonferroni correction with α = 0.05. ***p < 0.00009; n.s. not significant. **e**, Nf1-23a GTP hydrolysis by KRas is only inhibited by Zn. Hydrolysis was measured in the presence of 50 μM metal using a GTPase assay kit (Abcam) to detect the amount of phosphate ions (P_i_) in solution, with 3 independent replications. The concentration of P_i_ in each assay was normalized against both the buffer + metal and Nf1 + metal control. Error bars represent the mean ± s.e.m. of n = 3 independent assays of the same sample. Significance calculated by one-way ANOVA followed by pairwise 2-sided t-tests, applying Bonferroni correction with α = 0.05. **p = 0.0023; n.s. not significant.

The closed conformation of native Nf1 is stabilized by a cysteine- and histidine-coordinated transition metal binding site between the N-HEAT/ARM domain and the GRD-Sec14-PH linker L2 and is formed by the triade C1032, H1558 and H1576 (Fig. 2c), with the fourth coordination partner a solvent accessible water molecule (Fig. 3b). The tetrahedral site coordination is clear in map density and typical for Zn^2+^. The His_2_Cys motif presents the most preferred binding site coordination for Zn^2+^ in proteins^18^. After domain rotation to the open conformation, C1032 of the N-HEAT/ARM core is separated by 30 Å from the two histidine residues of the relocated GRD-Sec14-PH linker, and the metal binding site is lost (Fig. 2c). X-ray fluorescence scans confirmed the presence of Zn in native Nf1 (Extended Data Fig. 7c).

## Zn stabilizes the closed conformation

Upon addition of Zn to native Nf1, structural analysis revealed a stabilized Nf1 state with both protomers in the closed conformation and their Zn sites occupied. In contrast, Nf1 stripped with 1 mM EDTA destabilized the protein significantly, as shown by the higher void peak in the size exclusion chromatogram (Extended Data Fig. 7a). Zn-stabilized Nf1 yielded high-resolution maps, particularly of the GRD-Sec14-PH region, corroborating a stabilising role of Zn (Extended Data Figs. 2, 3). Ras-GAP activity assays (Fig. 3b, Extended Data Fig. 7d) confirmed that Zn-Nf1 shows concentration-dependent, significantly lower activity than the native, mixed-state Nf1. In contrast, 3d transition metal ions of Mn, Fe, Ni, Cu and Ca did not suppress Ras-GAP activity (Extended Data Fig. 7e).

## Exon 23a loop insert

The 63-nucleotide exon 23a inserts 21 amino acids into the Nf1 GRD between residues Q1370 and V1371, extending helix α56 by one turn before it loops back into helix α57, with residues A1380 to R1396 remaining disordered at the lysine-rich loop apex. Based on the SPRED1-GRD-Sec14-PH X-ray complex models^19^, no immediate direct interference of this flexible 23a insert is evident with either Ras or SPRED1 bound to the GRD (Fig. 3). Any structural role causal to reduced Ras-GAP activity is, therefore, likely indirect. However, in the open conformation, the 23a insertion loop points towards the membrane where access to the membrane-anchored Ras or to SPRED1 for Nf1 recruiting^13^ is crucial. Interference by the partly disordered, basic and hydrophilic 23a insertion could thus be contributing to diminished Ras-GAP activity of Nf1-23a^12^.

## Sec-14-PH lipid-binding domain

**Extended Data Fig. 8:**
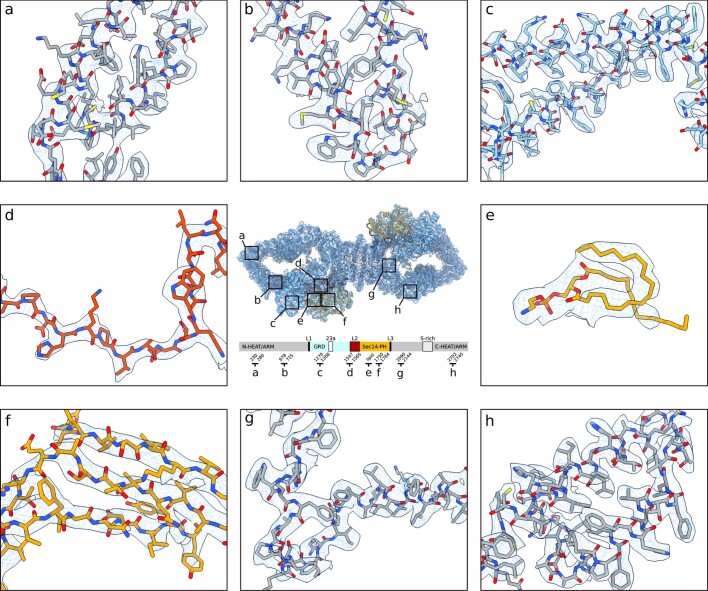
Quality of cryo-EM density maps. Different regions are shown starting from N- to C-terminus of Nf1. The labels of the panels correspond to regions and residue ranges in the central overview panel and the domain scheme. Coulomb potential density of **a**, the N-HEAT/ARM in the Nf1 tip region, **b**, the N-HEAT/ARM in the Nf1 core region, **c**, the GRD, **d**, the L2 linker between GRD and Sec14-PH, **e**, the lipid bound to Sec14-PH, **f**, resolved beta-strands of Sec14-PH, **g**, the C-HEAT/ARM in the Nf1 core region, **h**, the C-HEAT/ARM at the C-terminus.

In the closed protomer conformation, access to the hydrophobic core of the Sec14-PH domain that, in the X-ray models^20,21^, harbours a phosphatidylethanolamine representing a membrane lipid component, is largely blocked by the GRD. In Zn-Nf1 maps with 3.0 Å local resolution, the lipid could be modelled as phosphatidylethanolamine (Extended Data Fig. 8e). In the open state, the hydrophobic cavity of Sec14-PH is readily accessible and exposed. No major conformational rearrangements in the Nf1-23a Sec14-PH domain core relative to the X-ray models were observed (Fig. 3d).

## EM GRD domains differ from X-ray models

Although the X-ray models of GRD and Ras-bound GRD differ in detail (Fig. 3d), they are conformationally similar overall, with a modest backbone root-mean-square deviation (RMSD) of ~1.0 Å. The Nf1-23a GRD RMSD to the X-ray models and between open and closed form is around ~2.5 Å, but shifts of secondary structure elements up to ~7 Å occur (Fig. 3d). The well-defined linker L2 connecting the GRD with the Sec14-PH domain is unique to the cryo-EM model. In closed conformation, the GRD arginine finger R1276, critical for Ras binding^22,23^, is not accessible. Reorientation of the GRD in the open conformation exposes the arginine finger, which becomes accessible for Ras binding.

## SPRED1 binding to Nf1

**Fig. 4:**
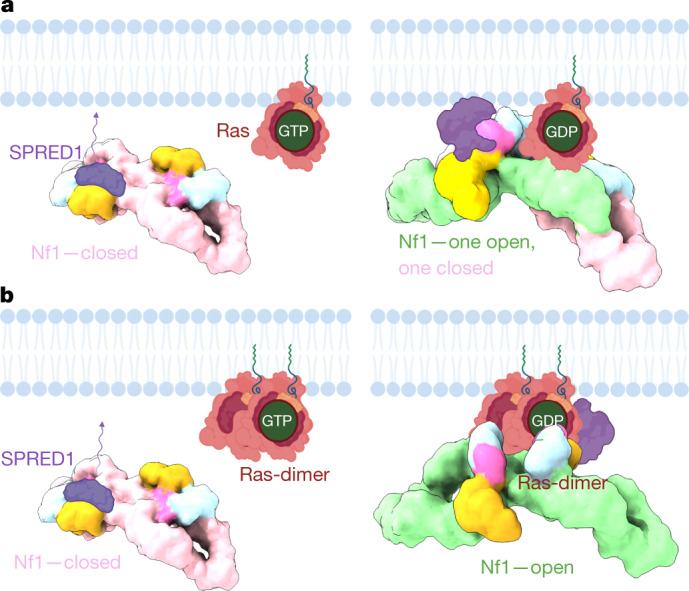
Postulated neurofibromin action. **a**, In the closed state, inactivated Nf1 cannot downregulate Ras. In the active, open Nf1 state, SPRED1 can bring Nf1 to the cell membrane where Nf1 can bind and downregulate monomeric Ras×GTP to Ras×GDP. The open Nf1 state could also regulate a Ras×GTP dimer. **b**, Hypothetical model of a not-observed Nf1 state with two partially open GRDs that would allow downregulation of one Ras×GTP dimer at the same time in one cycle.

SPRED1 recruits Nf1 by the GAPex domain from the cytosol to the membrane, where Nf1 can interact and downregulate Ras^14,24^. Mutations in SPRED1 were linked to the distinct rasopathy legius syndrome, related to NF1^25^. These diseases can be largely explained by impaired SPRED1-mediated membrane recruiting of Nf1. The structure reveals that, unlike Ras, SPRED1 can bind to both Nf1 conformations (Fig 3c), indicating that SPRED1 can bind cytosolic Nf1 also in its closed state and recruit it to the membrane. However, SPRED1 bound to the open, GAP-active conformer has a different orientation relative to the membrane than SPRED1 bound to the closed protomer (Fig 3c). It is possible that SPRED1 bound to the closed conformer suffices to recruit Nf1 to the membrane where the open conformer then downregulates Ras activity (Fig. 4a). The role of a second SPRED1 possibly bound to the open conformer remains unresolved.

## Implications for NF1 function

The full-length Nf1-23a dimer structures highlight the complexity of Nf1 function, pointing towards a network of allosteric regulatory effects. The Nf1 HEAT/ARM dimer framework is rigid in its core, providing a stable platform for the GRD-Sec14-PH domain rearrangement while retaining high mobility at the peripheral tips of the two lobes. The resulting structural complexity and involvement of massive rearrangements of the GRD and Sec14-PH domain against the core can explain the wide variety of disease-relevant mutations^4^.

The exact reason for the flexibility and dimerization is unknown but is likely required to trigger or facilitate transition between closed inactive and open active conformations. If both states are energetically similar, thermal motion might provide enough torque to trigger or support a transition between states. Active NTP-dependent mechanisms cannot be ruled out, but a nucleotide-binding pocket in the structure remains to be discovered. The role of the unobserved, mobile S-rich domain in triggering domain rearrangements remains unresolved.

Because both Nf1 protomers in the dimer cannot assume the open conformation at the same time (Fig. 3a), Ras-GAP regulation would have to occur with one Nf1 dimer regulating one Ras×GTP monomer or Ras×GTP dimer at the time (Fig. 4a). However, the existence of structurally different open states that could accommodate binding of a Ras×GTP dimer by two structurally different open conformer protomers is possible. Binding and regulation of Ras dimers by Nf1 remains an open line of investigation (Fig. 4b), and it is uncertain how many SPRED1 will bind in this process. For an isolated open–closed conformation Nf1 dimer, two SPRED1 domains could bind without interference, but their relative orientations differ, and it is unknown which configuration is causal in membrane recruiting. The Sec-14-PH domain might play a role as a membrane sensor and might impart additional regulatory function on Nf1. Lipid exchange function was established^20^ for Sec14-PH, indicating that Sec14-PH can sense the membrane environment before Nf1 transitions into the open state.

High levels of intracellular Zn (‘Zn wave’) were reported, dependent on calcium and MAPK signalling activity^26^, and the presence of a cysteine-coordinated Zn binding site^27^ suggests involvement of a secondary regulatory element in the Nf1 Ras-GAP activity.^26,27^  Zn stabilizes the auto-inhibited conformation of Nf1, and, consequently, Ras is upregulated, which, in turn, would lead to higher MAPK signalling. However, our concentration-dependent Zn assays indicate that, to suppress Nf1 activity, Zn concentrations must be significantly higher than presumed cytosolic steady-state concentrations. Given the complexity of the cellular Zn metallome and unknown spatiotemporal Zn concentrations^28^, we cannot presently draw firm conclusions about a regulatory role of Zn as a second messenger. Additional studies should clarify the exact role of Zn in the Ras signalling pathway, especially whether Nf1 can be regulated in vivo by Zn and how Zn release into the cytosol is triggered and regulated.

The dynamics of Nf1 might be additionally modulated by the many described, but not well-characterized, Nf1 interaction partners^4^. Regulation of Nf1 likely occurs in many layers, and our study sets the stage for research aiming to clarify the exact mechanism of Nf1 action and its related, multifaceted disease, NF1.

## Methods

### Cloning, expression and purification of human Nf1-23a

Full-length human Nf1-23a (Nf1 isoform 2, UniProt accession no. P21359), including an N-terminal strep tag followed by a TEV cleavage site, was designed and generated synthetically (GeneArt) and cloned into pACEBac1 baculovirus transfer vector^29^. Nf1-23a was expressed in *Sf21* insect cells for 2 d after baculovirus infection (Bac-to-Bac, Invitrogen). All subsequent steps were carried out at 4 ºC. Cells (~0.6 ml at ~1 million cells per ml) were harvested by centrifugation (1,000*g* for 10 min) and resuspended in 20 ml of lysis buffer containing 50 mM Tris pH 8.0, 300 mM NaCl, 2 mM DTT, 1 mg ml^−1^ of DNase I and a protease inhibitor cocktail tablet (EDTA-free, Sigma-Aldrich). Suspended cells were lysed by 20 strokes with a Dounce homogenizer on ice. Cell debris was removed by centrifugation (20,000*g* for 1 h), and the supernatant was filtered through a 0.8-µm polystyrene membrane (Millipore). The sample was loaded onto a Strep-Tactin column (5 ml), washed with 10 column volumes (CV) of wash buffer (50 mM Tris pH 8.0, 300 mM NaCl and 2 mM DTT), and the protein was eluted with 5 CV of wash buffer containing 5 mM desthiobiotin. The eluate was concentrated to ~5 mg ml^−1^ using a 100-kDa concentrator (Amicon Ultra, EMD Millipore) and run on a Superose 6 Increase 10/300 size-exclusion chromatography (SEC) GL column, equilibrated with gel filtration buffer (20 mM Tris pH 8.0, 300 mM NaCl and 2 mM DTT). Elution fractions containing Nf1-23a were pooled and concentrated to ~4 mg ml^−1^ using a 100-kDa concentrator (Amicon Ultra, EMD Millipore). Purified protein was flash-frozen in liquid nitrogen until further use.

For the second dataset containing Zn, freshly purified Nf1-23a at ~0.5 mg ml^−1^ was incubated for 90 min with a final concentration of 50 µM ZnCl_2_ before freezing grids. Sample preparation and data collection were carried out as described for native Nf1-23a.

To determine the stability of Nf1-23a in the absence of metals, the protein was purified in the presence of 1 mM EDTA throughout the preparation. This resulted in a higher void peak (~9 ml) in the final gel filtration column profile (Extended Data Fig. 7a), indicating that the protein is less stable in the absence of metals.

### Cloning, expression and purification of human KRas

Wild-type human KRas (amino acids 1–169, UniProt accession no. P01116) with amino terminal 6×His tag was purchased from Addgene and expressed overnight in *Escherichia coli* cells at 18 ºC. All subsequent steps were carried out at 4 ºC. Cells were harvested by centrifugation (3,000*g* for 20 min) and lysed twice by sonication on ice in a buffer containing 50 mM HEPES pH 8.0, 300 mM NaCl, 0.1 mM GTP (Sigma-Aldrich), 1 mM MgCl_2_, 2 mM TCEP, 1 mg ml^−1^ of DNase I and a protease inhibitor cocktail tablet. Cell debris was removed by centrifugation (20,000*g* for 1 h), and the supernatant was filtered through a 0.8-µm polystyrene membrane (Millipore). The sample was loaded onto a HisTrap column (5 ml), washed with 10 CV of wash buffer (50 mM HEPES pH 8.0, 300 mM NaCl, 1 mM MgCl_2_ and 20 mM imidazole), and the protein was eluted with 5 CV of elution buffer (50 mM HEPES pH 8.0, 300 mM NaCl, 1 mM MgCl_2_ and 200 mM imidazole). The eluate was supplemented with 0.1 mM GTP, concentrated to ~5 mg ml^−1^ using a 10-kDa concentrator (Amicon Ultra, EMD Millipore) and run on a HiLoad 16/60 Superdex 200 SEC GL column, equilibrated with gel filtration buffer (20 mM HEPES pH 8.0, 300 mM NaCl, 0.1 mM GTP, 1 mM MgCl_2_ and 2 mM DTT). Elution fractions containing KRas were pooled and concentrated to ~4 mg ml^−1^ using a 10-kDa concentrator (Amicon Ultra, EMD Millipore). Purified protein was flash-frozen in liquid nitrogen until further use.

### Cryo-EM sample preparation and data acquisition

Quantifoil R2/1 holey carbon grids (Au 300 mesh, Electron Microscopy Sciences) were glow-discharged for 60 s at 20 mA using a GloQube (Quorum) instrument. Purified Nf1-23a was thawed, centrifuged (14,000*g* for 5 min at 4 ºC) and diluted to ~0.5 mg ml^−1^ with gel filtration buffer. Protein was loaded into the freshly glow-discharged grids and plunge-frozen in LN_2_-cooled liquid ethane using a Vitrobot Mark IV (Thermo Fisher Scientific) with a blot force of −2 for 2.5 s. Temperature and relative humidity were maintained at 4 ºC and 100%, respectively. Grids were clipped and loaded into a 300-kV Titan Krios G2 microscope (Thermo Fisher Scientific, EPU 2.8.1 software) equipped with a Gatan BioQuantum energy filter and a K3 Summit direct electron detector (AMETEK). Grids were screened for quality control based on particle distribution and density, and images from the best grid were recorded. Micrographs were recorded at a nominal magnification of ×105,000, corresponding to a calibrated pixel size of 0.86 Å. The dose rate was 12 electron physical pixels per second, and images were recorded for 3.3 s divided into 40 frames, corresponding to a total dose of 40 electrons per Å^2^. Defocus range was set between −0.5 μm and −4 μm. Gain-corrected image data were acquired.

### Cryo-EM data processing

Extended Data Fig. 1 illustrates the data processing workflow for the native Nf1-23a dataset. The following pre-processing steps were performed with cryoSPARC Live v3.1.0 (ref. ^30^). Movie stacks were motion-corrected and dose-weighted using MotionCor2 v2.1.1 (ref. ^31^). Contrast transfer function (CTF) estimates for the motion-corrected micrographs were calculated with CTFFIND4 v4.1.13 (ref. ^32^). Poor-quality micrographs were discarded. Particles were initially picked with a blob-picker using subset-selected micrographs, and these were used for reference-free two-dimensional (2D) classification to generate picking templates. Auto-picking (using 23 of the representative 2D classes containing different orientations as templates) from 7,848 micrographs yielded ~1.4 million particles. An initial model was generated without imposing symmetry (C1) using stochastic gradient descent in cryoSPARC Live. Subsequent image processing was carried out with cryoSPARC v3.1.0 (ref. ^33^). Particles were classified with three-dimensional (3D) heterogenous refinement using four classes, resulting in 714,512 particles. Further classification using reference-free 2D classification with 100 classes yielded 582,742 particles. Particles were converted into a STAR file and input into RELION v3.1.1 (ref. ^34^) for further processing.

To generate a consensus reconstruction, particles were re-extracted in 470 pixels (1.7× binned) followed by 3D refinement with local angular search using the cryoSPARC map as the starting model. ‘Polished’ particle images, which were corrected for individual particle movements, were generated using aligned movie frames that were output from MotionCor2. 3D refinement on the polished particles, followed by CTF refinement and another round of 3D refinement, yielded a reconstruction to ~3.4 Å overall resolution with C1 symmetry.

Using the polished particles and the consensus reconstruction, signal subtraction was carried out on each lobe separately with reboxing to 235 pixels and recentring on the mask. 3D refinement followed by CTF refinement and another round of 3D refinement improved the reconstruction of each lobe to ~3.1 Å overall resolution with C1 symmetry (Extended Data Fig. 1).

To generate reconstructions for the different conformations of Nf1-23a, the unpolished 582,742 images were used in RELION v3.1.1. Particles were re-extracted in 400 pixels (2× binned) followed by 3D refinement. Polished particle images were first generated, followed by three rounds of 3D refinement and CTF refinement (without 4th-order aberrations), in an alternative manner. This yielded a reconstruction to ~3.5 Å overall resolution in C1 symmetry. To generate the closed (auto-inhibited) conformation reconstruction, signal subtraction was carried out on the particles using a mask on lobe 2, with reboxing to 200 pixels and recentring on the mask. After 3D refinement, 3D classification (with three classes) without alignment to generate a class with 130,329 particles representing the closed conformation was performed. Signals for these particles were reverted to the original unsubtracted images, and 3D refinement yielded the auto-inhibited reconstruction at overall resolution of ~4 Å (Extended Data Fig. 1).

One of the classes from the above 3D classification with 189,570 particles had a lobe in the open conformation. Another round of 3D classification (with three classes) without alignment yielded a class with 51,951 particles that had a better resolution for the open conformation. Signals for these particles were reverted to the original, and 3D refinement yielded the open reconstruction at an overall resolution of ~4.8 Å (Extended Data Fig. 1).

The 582,742 particles polished above with an overall resolution of ~3.5 Å were used to improve the resolution on the GRD-Sec14-PH domain. A mask was generated on this region in lobe 1, and the signal outside of the mask was subtracted with reboxing to 200 pixels and recentring on the mask. After 3D refinement, 3D classification was performed (with three classes) without alignment to generate a class with 100,684 particles that had a better overall resolution. 3D refinement on these particles yielded a reconstruction to ~3.5 Å resolution (Extended Data Fig. 1).

Extended Data Fig. 2 illustrates the data processing workflow for the dataset from Zn-Nf1. The pre-processing steps were performed with cryoSPARC Live v3.1.0 as described above. Auto-picking (using 33 of the representative 2D classes containing different orientations as templates) from 18,882 micrographs yielded ~2.3 million particles. Subsequent image processing was carried out with cryoSPARC v3.1.0. Particles were classified with 3D heterogenous refinement using four classes, resulting in 1,046,333 particles. Further classification using reference-free 2D classification with 100 classes yielded 843,857 particles. Particles were refined to ~3.6 Å resolution with homogenous refinement and re-extracted in 384 pixels (1.56× binned) using the refined coordinates. Further refinement of the particles using non-uniform refinement yielded a reconstruction to ~3.3 Å resolution. The refined particles were converted into a STAR file and input into RELION v3.1.1 for further processing.

To generate a consensus reconstruction, particles were re-extracted in 400 pixels (1.5× binned) followed by 3D refinement with local angular search using the consensus map generated from the first dataset as the starting model. After CTF refinement (without the 4th-order aberrations) and 3D refinement, particle images were polished to correct for individual particle movements, using aligned movie frames that were output from MotionCor2. 3D refinement on the polished particles yielded a reconstruction to ~3.3 Å overall resolution with C1 symmetry. However, there was some anisotropy in the reconstructed map, likely due to the presence of different conformations of the particles. To improve the reconstruction, the polished particles were classified using 3D classification (with five classes) without alignment. One class containing 300,087 particles had GRD-Sec14-PH domains on both lobes that were well defined. 3D refinement, followed by CTF refinement (without 4th-order aberrations) and 3D refinement on these particles yielded a consensus reconstruction to ~3.3 Å, which did not show anisotropy artefacts.

Using the 300,087 particles from the above 3D classification, signal subtraction was carried out on each lobe separately with reboxing to 270 pixels and recentring on the mask, and the subtracted particles were then combined. 3D refinement followed by CTF refinement and another round of 3D refinement using a mask on the core improved the reconstruction in this region of each lobe to ~2.9 Å overall resolution with C1 symmetry (Extended Data Fig. 2). Using another mask for the tip of each lobe improved the reconstruction in this region for each lobe to ~3.1 Å overall resolution with C1 symmetry.

To improve the resolution for the GRD-Sec14-PH domain on each lobe, the 843,857 polished particles were used to subtract signal outside of this domain in each lobe separately, with reboxing to 270 pixels and recentring on the mask. 3D classification (three classes) without alignment was carried out on each GRD-Sec14-PH domain. The GRD-Sec14-PH domain in each lobe contained one class (356,665 particles in lobe 1 and 309,370 particles in lobe 2), which had a better resolution. These classes were combined, and, subsequently, 3D refinement, CTF refinement and another round of 3D refinement were performed to yield a reconstruction for the GRD-Sec14-PH domain to ~3.0 Å resolution with C1 symmetry.

All final reconstructions were sharpened using either RELION post-processing or DeepEMhancer^35^. The local resolution estimations in Extended Data Fig. 3 were performed using Resmap v1.1.4 (ref. ^36^).

### Model building and refinement

A partial starting model for the Nf1-23a core was built ab initio by Buccaneer v1.6.10 (ref. ^37^) and manually completed and real-space refined into the highest resolution map (3.2 Å) with Coot 0.9.4.1 (refs. ^38,39^). The GRD of 1nf1 (ref. ^17^) and the 2e2x Sec14-PH domain^20^ were rigid body fitted using Chimera^40,41^ into the best GRD-Sec14-PH map and manually corrected and completed by real-space refinement in Coot. Additional linker regions were built manually in Coot. The core model as well as the GRD-Sec14-PH model were real-space refined with PHENIX v1.19-4092 real-space-refine^42^ into the corresponding domain maps. The resulting refined models were then fitted into the consensus map using UCSF Chimera v1.15 and refined by PHENIX real-space-refine. Similarly, for the closed conformation, the model of the core and the model of the GRD-Sec14-PH domains were fitted into the density and real-space refined in Coot, followed by connecting the different domains of the model. For the open conformation, the GRD and Sec14-PH domains were rigid body fitted in Chimera followed by manual modelling of the linker regions in Coot. The closed and opened state models were refined by PHENIX real-space-refine using maps generated by DeepEMhancer^35^. All structure models were validated using the PDB validation service^43^. Dimer interfaces and buried surface areas were computed with ePISA v1.52 (ref. ^44^). Data collection, refinement and model quality statistics are summarized in Extended Data Table 1.

For the Zn-stabilized structure, the Nf1 closed state of the native dataset was rigid body fitted into a composite map that was generated with PHENIX combine-focused-maps out of all high-resolution maps for the different parts (tip, core and GAP-Sec14-PH region). The model was real-space refined using Coot and refined with PHENIX real-space-refine.

PDB deposition codes of X-ray structure models used in analysis and modelling were 2d4q^45^, 2e2x^20^, 3peg, 3pg7, 3p7z^21^, 1nf1 (ref. ^17^), 6ob2, 6ob3 (ref. ^46^), 6v65 and 6v6f^19^ (Supplementary Table 1).

### GAP activity assays

Assays for GAP-stimulated GTP hydrolysis were performed using a GTPase assay kit (Abcam), according to the manufacturer’s instructions. The assay uses a malachite photometrically green reagent to measure the concentration of phosphate ions (P_i_) in solution. In brief, KRas (wild-type) was first buffer exchanged into buffer A (50 mM HEPES pH 8.0 and 300 mM NaCl) to remove any excess of free nucleotides and P_i_ and then added to white opaque 96-well plates (Corning) at a final concentration of 0.5 μM. GTPase reactions were initiated by the addition of Nf1 (buffer exchanged into buffer A) at a final concentration of 0.5 μM (final volume 100 μl with 0.5 mM GTP and 2.5 mM MgCl_2_ added to the assay buffer). Samples were incubated for 90 min at room temperature, and the absorbance was measured at 600 nm using a SpectraMax i3x plate reader (Molecular Devices). To test the effect of divalent cations, ZnCl_2_, MnCl_2_, FeCl_2,_ CaCl_2_, CuCl_2_ and NiCl_2_ were added to each assay buffer at a final concentration of 50 µM. The effect of different concentration of ZnCl_2_ (10–100 µM) on the rate of Nf1-23a GTP hydrolysis by KRas was also measured. A standard curve of absorbance for known concentrations of P_i_ was generated and used to estimate the concentration of P_i_ in each sample. Each assay was repeated by three, five or eight independent experiments. Data are expressed as the mean ± s.e.m. Significance was calculated by one-way ANOVA followed by pairwise two-sided *t*-tests, applying Bonferroni correction^47^. The high concentration of protein used in the assays and the long incubation periods were necessary to measure µM concentrations of P_i_ in a relatively large volume in each assay. Raw assay data and analysis are available in Supplementary Data 1.

### Total reflection X-ray fluorescence

The elemental nature of the bound metal cation in native Nf1-23a, purified without any Zn addition, was determined using total reflection X-ray fluorescence (TXRF) analysis on a Bruker PICOFOX S2 instrument. Both sample and buffer were measured in the presence of a gallium internal standard at 2 mg l^−1^ added to the samples (1:1, v/v) before the measurements. TXRF spectra were analysed using the Bruker PICOFOX Super Bayes Quantification software provided with the spectrometer. The highest metal peak was clearly identified as Zn (Extended Data Fig. 7c).

### Sequence alignment

Homologous sequences of established and candidate HEAT/ARM proteins were identified through PSI-BLAST searches in the MPI Bioinformatics Toolkit (three iterations, default parameters)^48^. Sequences with unusual insertions and/or deletions were removed, as were sequences that aligned very poorly. Each set of retrieved sequences, as well as the sequences for Nf1-23a residues 1–1,193 and 1,850–2,839, were submitted for alignment to the threefold prediction server HHpred (https://toolkit.tuebingen.mpg.de/tools/hhpred)^49,50^. The server was run with default parameters with three iterations of global alignment against PDB_mmCIF70_3_Mar and PDB_mmCIF30_3_Mar databases. The alignments with the highest probabilities (>93%) and the lowest *E* values (<0.25) were used for representation in Extended Data Fig. 4, with multiple sequence alignments done in Jalview^51^ using Clustal W colouring^52^.

Extended Data Fig. 6 with secondary structure depiction of Nf1 was generated with https://espript.ibcp.fr (ref.^53^). Figure panels were created with UCSF Chimera^40^ and UCSF ChimeraX^41^. All graphs were made with Prism v8.3.0 (GraphPad Software, www.graphpad.com) and Biorender (https://biorender.com/).

### Reporting summary

Further information on research design is available in the Nature Research Reporting Summary linked to this paper.

## Online content

Any methods, additional references, Nature Research reporting summaries, source data, extended data, supplementary information, acknowledgements, peer review information; details of author contributions and competing interests; and statements of data and code availability are available at 10.1038/s41586-021-04024-x.

### Supplementary information

Supplementary Information This file contains Supplementary Fig. 1 (the uncropped blots) and Supplementary Table 1, which shows the compilation of Nf1 domain X-ray structure models.

Reporting Summary

Supplementary Data 1 GAP assay data and statistical analysis. Original assay data measurements and analysis are described in the Methods, ‘GAP activity assays’ section.

Supplementary Video 1 Morphing video of Nf1 from closed to open state. The video shows the transition from the closed, auto-inhibited, inactive state of Nf1-23a to its open, active state. Content was created with Chimera X, edited in Microsoft Movie Maker.

## Figures and Tables

**Extended Data Table 1:**
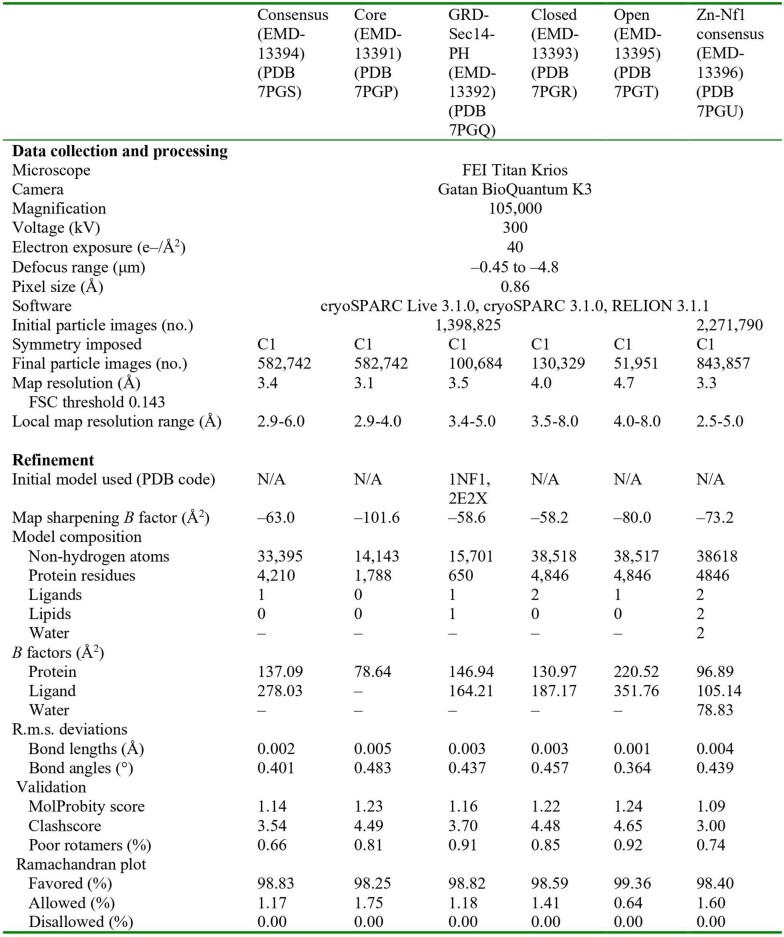
Cryo-EM data collection, model refinement, and validation statistics summary

Three local reconstructions without fitted models from Extended Data Fig. 2 have also been deposited with the following EMDB codes: EMD-13397 (Zn-Nf1 tip), EMD-13398 (Zn-Nf1 core), EMD-13399 (Zn-Nf1 GRD-Sec14-PH).

## Data Availability

All cryo-EM density maps, half maps, masks, Fourier shell correlation curves and composite maps were deposited into the Electron Microscopy Data Bank (https://www.ebi.ac.uk/pdbe/emdb/) under accession codes EMD-13394, EMD-13391, EMD-13392, EMD-13393, EMD-13395 and EMD-13396. The corresponding model coordinates were deposited in the Protein Data Bank (https://www.ebi.ac.uk/pdbe) under accession codes 7PGS, 7PGP, 7PGQ, 7PGR, 7PGT and 7PGU. Local map reconstructions without fitted models were deposited under codes EMD-13397 (Zn-Nf1 tip), EMD-13398 (Zn-Nf1 core) and EMD-13399 (Zn-Nf1 GRD-Sec14-PH). All assay data are supplied as Supplementary Data.
