# Supplementary material for: The structure of neurofibromin isoform 2 reveals different functional states
Source: Nature. 2021 Oct 27;599(7884):315–9. doi: 10.1038/s41586-021-04024-x (PMC8580823; doi:10.1038/s41586-021-04024-x)
Supplement: Supplementary file 1 — This file contains Supplementary Fig. 1 (the uncropped blots) and Supplementary Table 1, which shows the compilation of Nf1 domain X-ray structure models. [file 41586_2021_4024_MOESM1_ESM.pdf]

nature portfolio

https://doi.org/10.1038/s41586-021-04024-x

Supplementary information

The structure of neurofibromin isoform 2 reveals different functional states

In the format provided by the authors and unedited

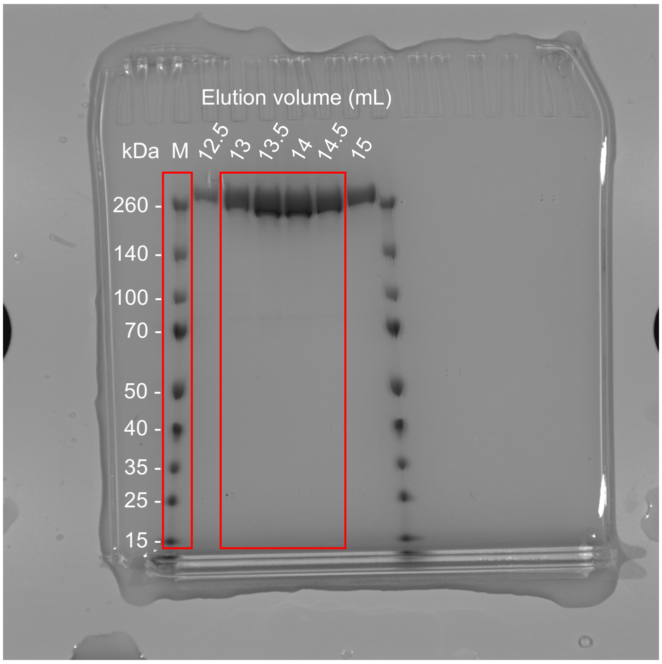

Control

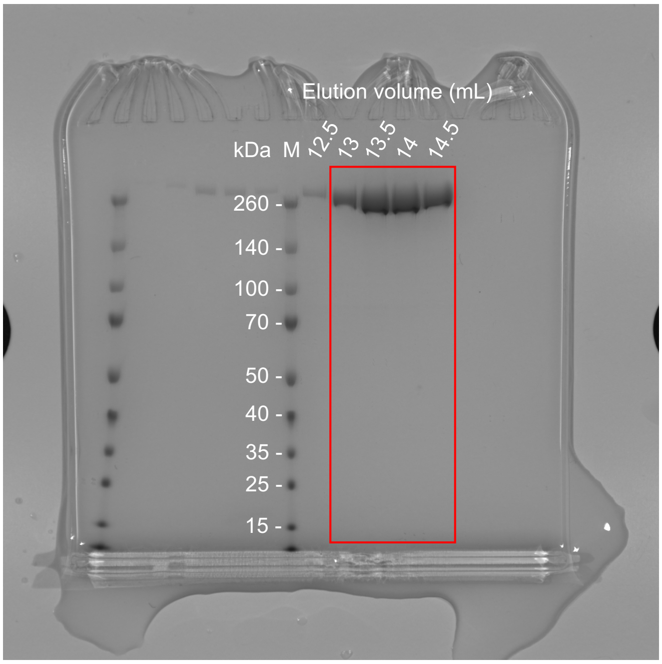

EDTA

Supplementary Figure 1 | Uncropped SDS-PAGE gels from Extended Data Figure 7b. The two proteins were purified in parallel and run on different SDS-PAGE gels. The red rectangles show the cropping locations.

Supplementary Table 1 | Compilation of Nf1 domain X-ray structure models. X-ray crystal structures are available of the lipid binding Sec14-PH domain and mutants; the GRD; and GRD with KRAS and mutants, and ternary complexes with KRAS, GRD and SPRED1.Residue numbers in parenthesis correspond to Nf1-23a sequence (21 residue insertion after residue Q1370).

PDB_Id.	Resolution	Protein(s)	Nf1 residues, ligands	Remarks	Reference
2d4q	2.3	Sec14-PH	1560-1816 (1581-1837)<br>OXN TritonX-100<br>POP pyrophosphate	A, B dimer	45
2e2x	2.5	Sec14-PH	TAG-1545-1816 (1566-1837)<br>PEV phosphatidylethanolamine<br>POP pyrophosphate	A, B dimer	20
3peg	2.53	Sec14-PH insertion mutant	TAG-1545-1816 (1566-1837)<br>PEV phosphatidylethanolamine	1699D-1712D insertion at 1712-1713	21
3pg7	2.19	Sec14-PH L1771 deletion mutant	1560-1815 (1581-1837)<br>PTY phosphatidylethanolamine<br>POP pyrophosphate	A, B dimer<br>L1771 deletion	21
3p7z	2.65	Sec14-PH I1584V mutant	TAG-1545-1816 (1566-1837)<br>PEV phosphatidylethanolamine<br>POP pyrophosphate	A, B dimer<br>I1584V mutant	21
1nf1	2.5	GRD	1198-1530<br>Missing 1198-1205, 1305-1330, 1404-1411, 1464-1448, 1504-1512	No structure factors available	17
6ob2	2.85	GRD + KRas	1-169, 1209-1463 (1484)<br>GNP	KRas A, C; GRD B, D	46
6ob3	2.1	GRD + G13D-KRas	1-169, 1209-1463 (1484)<br>GNP	KRas A, C; GRD B, D,	46
6v65	2.76	SPRED1+GRD+Ras	13-125, 1203-1530 (1551), 1-169<br>GNP	A, B, C chains	19
6v6f	2.54	SPRED1+GRD+ Q61LRas	13-125, 1203-1530 (1551), 1-169<br>GNP	A, B, C chains	19
