# Supplementary material for: The structure of neurofibromin isoform 2 reveals different functional states
Source: Nature. 2021 Oct 27;599(7884):315–9. doi: 10.1038/s41586-021-04024-x (PMC8580823; doi:10.1038/s41586-021-04024-x)
Supplement: Supplementary file 2 — Reporting Summary [file 41586_2021_4024_MOESM2_ESM.pdf]

nature research

Corresponding author(s): Bernhard Rupp (bernhard.rupp@i-med.ac.at)
Marta Carroni (marta.carroni@scilifelab.se)

Last updated by author(s): Aug 15, 2021

Reporting Summary

Nature Research wishes to improve the reproducibility of the work that we publish. This form provides structure for consistency and transparency in reporting. For further information on Nature Research policies, see our Editorial Policies and the Editorial Policy Checklist.

Statistics

For all statistical analyses, confirm that the following items are present in the figure legend, table legend, main text, or Methods section.

n/a	Confirmed	
☐	☒	The exact sample size (*n*) for each experimental group/condition, given as a discrete number and unit of measurement
☐	☒	A statement on whether measurements were taken from distinct samples or whether the same sample was measured repeatedly
☐	☒	The statistical test(s) used AND whether they are one- or two-sided<br>*Only common tests should be described solely by name; describe more complex techniques in the Methods section.*
☐	☒	A description of all covariates tested
☐	☒	A description of any assumptions or corrections, such as tests of normality and adjustment for multiple comparisons
☐	☒	A full description of the statistical parameters including central tendency (e.g. means) or other basic estimates (e.g. regression coefficient) AND variation (e.g. standard deviation) or associated estimates of uncertainty (e.g. confidence intervals)
☐	☒	For null hypothesis testing, the test statistic (e.g. *F*, *t*, *r*) with confidence intervals, effect sizes, degrees of freedom and *P* value noted<br>*Give P values as exact values whenever suitable.*
☒	☐	For Bayesian analysis, information on the choice of priors and Markov chain Monte Carlo settings
☒	☐	For hierarchical and complex designs, identification of the appropriate level for tests and full reporting of outcomes
☒	☐	Estimates of effect sizes (e.g. Cohen's *d*, Pearson's *r*), indicating how they were calculated

Our web collection on statistics for biologists contains articles on many of the points above.

Software and code

Policy information about availability of computer code

Data collection	EPU 2.8.1, Thermo Fisher Scientific
Data analysis	cryoSPARC Live v3.1.0, cryoSPARC v3.1.0, Relion 3.1.1, Motioncor2 2.1.1, Ctffind4 4.1.13, DeepEMhancer, Resmap v1.1.4, Buccaneer 1.6.10, Phenix 1.19-4092, COOT 0.9.4.1, ePISA v1.52

For manuscripts utilizing custom algorithms or software that are central to the research but not yet described in published literature, software must be made available to editors and reviewers. We strongly encourage code deposition in a community repository (e.g. GitHub). See the Nature Research guidelines for submitting code & software for further information.

Data

Policy information about availability of data

All manuscripts must include a data availability statement. This statement should provide the following information, where applicable:

- Accession codes, unique identifiers, or web links for publicly available datasets
- A list of figures that have associated raw data
- A description of any restrictions on data availability

All Cryo-EM density maps, half maps, masks, FSC curves and composite maps were deposited into the Electron Microscopy Data Bank (https://www.ebi.ac.uk/pdbe/emdb/), accession codes EMD-13394, EMD-13391, EMD-13392, EMD-13393, EMD-13395, EMD-13396. The corresponding model coordinates were deposited with the Protein Data Bank (https://www.ebi.ac.uk/pdbe), accession codes 7PGS, 7PGP, 7PGQ, 7PGR, 7PGT, 7PGU. Local map reconstructions without fitted models were deposited with codes EMD-13397 (Zn-Nf1 tip), EMD-13398 (Zn-Nf1 core), EMD-13399 (Zn-Nf1 GRD-Sec14-PH). All assay data supplied as Supplementary Data.

Field-specific reporting

Please select the one below that is the best fit for your research. If you are not sure, read the appropriate sections before making your selection.

☒ Life sciences ☐ Behavioural & social sciences ☐ Ecological, evolutionary & environmental sciences

For a reference copy of the document with all sections, see nature.com/documents/nr-reporting-summary-flat.pdf

Life sciences study design

All studies must disclose on these points even when the disclosure is negative.

Sample size	No Sample size calculations were performed. For cryo-EM samplesFrom 7,848 micrographs, 1,4 million particles were picked, 714,512 particles used in the final reconstruction, around 500,000 particles in 3D classes. This is standard procedure in cryoEM, and sample size was sufficient to achieve 3.3 A overall resolution. Sample size for independent supporting assay measurements between N=3 and N=8.
Data exclusions	No data were systematically excluded.
Replication	Cryo-EM single particle analysis averages independent particle observations, with around 500 000 particles contributing to each 3D class. Assay data were replicated multiple times from the same samples as described.
Randomization	All vitrified cryo-EM particles used for structure determination adopt random orientations on EM grids. Assignment of particles into random half data sets is automatically performed during 3D reconstruction by Relion 3.1.1. No other experiments involve or do require randomization.
Blinding	Blinding is not applicable for this study because no group allocation was applied or required.

Reporting for specific materials, systems and methods

We require information from authors about some types of materials, experimental systems and methods used in many studies. Here, indicate whether each material, system or method listed is relevant to your study. If you are not sure if a list item applies to your research, read the appropriate section before selecting a response.

Materials & experimental systems

n/a	Involved in the study	
☒	☐	Antibodies
☐	☒	Eukaryotic cell lines
☒	☐	Palaeontology and archaeology
☒	☐	Animals and other organisms
☒	☐	Human research participants
☒	☐	Clinical data
☒	☐	Dual use research of concern

Methods

n/a	Involved in the study	
☒	☐	ChIP-seq
☒	☐	Flow cytometry
☒	☐	MRI-based neuroimaging

Eukaryotic cell lines

Policy information about cell lines

Cell line source(s)	Gibco Sf21 cells (Thermo Fisher 11497013) in Sf-900 II medium, used only for Nf1-23a expression. No experiments generating data were performed with these cells.
Authentication	Cell lines were not authenticated in-house.
Mycoplasma contamination	Cell lines were not tested for mycoplasma contamination.
Commonly misidentified lines (See ICLAC register)	No commonly misidentified cell lines were used.
